# Research to meet the needs of the NHS: a review of published NIHR HTA clinical trials

**DOI:** 10.1186/1745-6215-12-S1-A86

**Published:** 2011-12-13

**Authors:** Amanda Young, Ruairidh Milne, James Raftery, Louise Dent, David Turner, Peter Davidson, Andrew Cook

**Affiliations:** 1 University of Southampton’s Wessex Institute, Southampton, SO16 7NS, UK; 2 NIHR, Evaluation, Trials and Studies Coordinating Centre (NETSCC), University of Southampton, Southampton, SO16 7NS, UK; 3 University of Southampton Clinical Trials Unit, Southampton General Hospital, Southampton, SO16 6YD, UK

## Background

The NIHR HTA programme was established in 1993 with the aim of being ‘needs-led’. This was achieved through commissioning trials to address questions specified by the programme rather than by applicants. At the time this was an unusual funding model and the programme has developed experience that is now globally significant.

There is dissatisfaction worldwide with the focus of much existing clinical research (the arrival in the USA of Comparative Effectiveness Research is one attempt to address this) and many trials focus on outcomes that are of little relevance to patients [1]. However, apart from early work reviewing the sources of topics suggested to the HTA programme [2], there has been no systematic attempt to review the programme’s success in being needs-led.

## Objectives

The establishment of a database of metadata for HTA trials allowed us to examine the experience from a long series of published HTA trials. We therefore set out to review how far trials funded by the NIHR HTA programme were indeed needs-led by looking at:

• The source of the original suggestion

• The priority given by the programme to the research

• The patient-relevance of the primary outcome

## Methods

The study used a variety of methods to assess the extent to which HTA trials published up to March 2011 were needs-led (n=112). The sources of topic identification and the prioritisation methods were examined over a 10 year period. The type of primary outcome measure reported during the commissioning stage, research protocol and HTA monograph were assessed using Gandhi *et al.* (2008) [1] classification list.

## Results

*The source of the original suggestion:* 77/112 trials (69%) addressed questions that came out of the widespread consultation, a mix of postal and online questionnaires of managers, clinicians and patient and professional groups. Majority of the remaining trials were from systematic reviews (22%, 25/112).

*The priority given by the programme to the research:* 58/84 had been recommended as ‘must commission’ priorities by the HTA programme, meaning that they would be advertised and if that failed to result in funding, further work would be done to ensure that they were taken forward.

*The patient-relevance of the primary outcome:* Three quarters of trials addressed patient relevant outcomes (75%, 84/112). This compares with 46% and 45% in previous studies [1], [3].

**Table 1:** Summary data

Description	No (%)
Sources used to identify research suggestions:	
Widespread consultation	77 (68.8)
Systematic review / DARE review	25 (22.3)
Horizon Scanning Centre	3 (2.7)
Reconsidered / recycled topics	5 (4.5)
No data available	2 (1.8)

Priority given by the programme to the research:	
(up to and including publication date 1999)	
Recommended for commissioning – must commission	58 (51.8)
Recommended for commissioning	22 (19.6)
Category unknown (FT and NSCAG)	4 (3.6)
(Post 1999)	
Commissioning requested by PSG	27 (24.1)
Direct commissioning in priority area	1 (0.9)

Actual type of primary outcome reported in the monograph:	
Patient important (including those with additional outcomes)	84 (75.0)
Surrogate	7 (6.3)
Physiological / laboratory	1 (0.9)
Other	18 (16.1)
No information available / unable to judge primary outcome	2 (1.8)

## Conclusions

These analyses suggest that the first 112 published HTA trials can indeed claim to be meeting the information needs of the NHS. Further work is required to compare these results with elsewhere and to develop more robust measures for the future.
